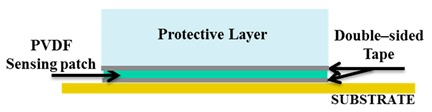# Validation of Screen-Printed Electronic Skin Based on Piezoelectric Polymer Sensors

**DOI:** 10.3390/s20041160

**Published:** 2020-02-20

**Authors:** Hoda Fares, Yahya Abbass, Maurizio Valle, Lucia Seminara

**Affiliations:** 1 Department of Naval, Electrical, University of Genoa, Electrical, Electronic and Telecommunications Engineering, via Opera Pia 11A, 16145 Genoa, Italy; hoda.fares@edu.unige.it (H.F.); YAHYA.ABBASS@edu.unige.it (Y.A.); maurizio.valle@unige.it (M.V.); 2 Ecole Doctorale des Sciences et de Technologie EDST, Lebanese University, Al Hadath, 1003 Beirut, Lebanon

**Keywords:** P(VDF-TrFE) sensors, screen printed sensor technology, technology validation, electronic skin, skin characterization, tactile sensing, prosthetics

## Abstract

This paper proposes a validation method of the fabrication technology of a screen-printed electronic skin based on polyvinylidene fluoride-trifluoroethylene P(VDF-TrFE) piezoelectric polymer sensors. This required researchers to insure, through non-direct sensor characterization, that printed sensors were working as expected. For that, we adapted an existing model to non-destructively extract sensor behavior in pure compression (i.e., the d_33_ piezocoefficient) by indentation tests over the skin surface. Different skin patches, designed to sensorize a glove and a prosthetic hand (11 skin patches, 104 sensors), have been tested. Reproducibility of the sensor response and its dependence upon sensor position on the fabrication substrate were examined, highlighting the drawbacks of employing large A3-sized substrates. The average value of d_33_ for all sensors was measured at incremental preloads (1–3 N). A systematic decrease has been checked for patches located at positions not affected by substrate shrinkage. In turn, sensor reproducibility and d_33_ adherence to literature values validated the e-skin fabrication technology. To extend the predictable behavior to all skin patches and thus increase the number of working sensors, the size of the fabrication substrate is to be decreased in future skin fabrication. The tests also demonstrated the efficiency of the proposed method to characterize embedded sensors which are no more accessible for direct validation.

## 1. Introduction

Electronic skin (e-skin) is a touch-sensitive, electronic system that incorporates functional and structural materials coupled to a suitable electronic interface for sensor signal acquisition. Tactile data processing algorithms might provide information about contact properties (e.g., contact force [1] or contact shape [2]), given properties of the contact object (such as surface texture [3], object shape [4]), or contact events (e.g., discrimination between touch modalities [5]), to cite some examples. Artificial skin systems are implemented in a wide range of applications, such as robotics, prosthetics and teleoperation systems [6,7,8].

As the functional properties of the electronic skin mostly depend on the sensor type, it is worth focusing on the sensor itself. Various tactile sensors have been developed, like piezoelectric, piezoresistive, capacitive, optical, electromagnetic, ultrasonic, etc. [6]. The development of tactile sensors based on piezoelectric polymers has been extensively investigated in recent years due to their interesting features. 

They exhibit high sensitivity, fast dynamic response and a large operating frequency range (from <1 Hz to 1 KHz), covering the whole frequency bandwidth of human skin mechanoreceptors [7]. Drawbacks of these materials are their poor temperature stability and their inability to measure static forces [8].

Different piezoelectric materials, such as quartz single crystals, ceramics and polymers, have been used to fabricate piezoelectric tactile sensors. Polymer materials, especially polyvinylidene fluoride (PVDF) and its (TrFE) Trifluoroethylene copolymers, exhibit ultra-sensitivity, flexibility and piezo, pyro and ferroelectric stability. Moreover, they have been proven to be good candidates for flexible tactile sensors, suitable for dynamic tactile sensing, and to be integrated into artificial electronic skin [7].

Regtien et al. [9] presented the advantages of P(VDF-TrFE) as tactile sensors, and Khan et al. [10] demonstrated fully screen-printed tactile P(VDF-TrFE) sensor arrays for robotic applications. Hsu et al. proved the strain sensitivity of PVDF-arrays on flexible substrates [11], and Tien et al. exploited the sensing multimodality with P(VDF-TrFE) gated OFETs for the simultaneous detection of pressure and temperature [12]. In general, the cross-sensitivity between temperature and pressure sensing in ferroelectrics (therefore also in PVDF) can become an issue; hence, a separation of the piezo- and pyroelectric effects may be advantageous [13]. 

PVDF is a semi-crystalline polymer synthesized by the polymerization of the H_2_C=CF_2_ monomer. Its copolymer, Poly (vinylidene fluoride trifluoroethylene) or P(VDF-TrFE), is a ferroelectric material that does not need to undergo the mechanical stretching of the molecular chains along one of the transversal axes, leading to easier fabrication. Different fabrication technologies have been reported for P(VDF-TrFE)-based sensors, such as spin coating, electrospinning, sol-gel, chemical vapor deposition, micromachined mold transfer and inkjet printing [14]. The frequently used techniques, such as spin coating and inkjet printing, have limitations of process speed and overlay registration accuracy in multilayered structures. Despite the high lateral resolution, the patterning of large areas through ink-jet printing requires the repeated deposition of droplets, which often results in a nonuniform layer thickness and edges. In addition, the patterning of P(VDF-TrFE) after spin coating requires photolithography, which leads to an increased complexity of the manufacturing process. The cost-effectiveness and faster fabrication of sensors over large areas indeed make screen-printing a very attractive technique [15]. 

Hoda et al. recently developed fully screen-printed tactile sensing arrays (in the following: sensing patches) based on P(VDF-TrFE) piezoelectric polymers for prosthetic applications [16], where arrays of piezoelectric polymer sensors provided of their metal contacts have been screen-printed on a transparent, plastic foil. The same fabrication process has been used to design and fabricate ad-hoc sensing patches to be mounted over two different systems, i.e., an assistive sensorized glove and the Michelangelo prosthetic Hand by Ottobock [17]. 

The focus of this study is the validation of the manufacturing technology, i.e., ascertaining that these printed sensors are working as expected. Characterizing sensor behavior directly would be a quite complex, lengthy, risky and hardly reproducible process. In addition, direct contact of the indenter with the sensor would have various shortcomings: (1) the contact would hardly be uniformly distributed, as both the indenter and sensor surfaces have natural roughness; (2) the contact surface could then not be precisely determined; (3) the direct indenter contact leads to sensor damage. Therefore, an indirect procedure is proposed here: It requires the integration of a protective layer on top of the sensing patch, giving rise to what we call the skin patch. As sensors are embedded into the mechanical structure of the skin, a model is needed to relate the applied force to sensor charge response, accounting for stress transmission through the cover layer. The reference for this approach is a validated model of analogous skin structure based on a rigid substrate, PVDF piezoelectric polymer sensors and the same (elastic) protective layer [18]. 

In detail, the present paper first reports the experimental setup and procedures which allow for a fast characterization of piezoelectric sensors embedded into an elastic layer and working in thickness mode (i.e., pure compression mode). For that, direct compression tests have been replaced by indentation tests over the skin surface, performed continuously over the whole frequency range of interest for tactile applications (<1 Hz–1 kHz). The model cited in the previous paragraph [18] has thus been used to estimate the d_33_ piezoelectric coefficient of each sensor from the measure of both a basic mechanical action at the skin surface and sensor charge, meaning P(VDF-TrFE) sensor electromechanical characterization. Finding d_33_ values aligned with expected values from the literature in turn validates each sensor and the skin fabrication technology. Finally, a short way to characterize future e-skin systems is provided. 

The paper is organized as follows: Section 2. presents the materials and methods, briefly illustrating the electronic skin design and technology, the reference skin model and the experimental setup. The results related to the validation of screen-printed sensing patches are reported in Section 3. Finally, our discussion and conclusive remarks are given in Section 4 and Section 5. 

## 2. Materials and Methods

### 2.1. Electronic Skin Design And Technology

#### 2.1.1. Screen-Printed Sensing Patches Based on Piezoelectric Polymers

**Figure 1:**
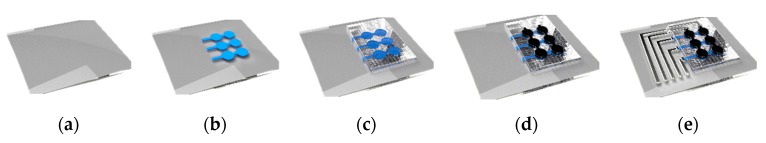
Illustration of the manufacturing process flow of printed ferroelectric sensor arrays based on polyvinylidene fluoride-trifluoroethylene P(VDF-TrFE) repeated units (reprinted with permission from JOANNEUM RESEARCH (JNR)). (**a**) Substrate; (**b**) Bottom Electrodes; (**c**) Active sensors based on P(VDF-TrFE) film + Top electrodes (1); (**d**) Top electrodes (2); (**e**) Connecting lines.

Fully screen-printed, flexible sensing patches based on P(VDF-TrFE) piezoelectric polymer sensors have been fabricated by JOANNEUM RESEARCH [19] (in the following, *JNR*). They patented a low-temperature, sol-gel based synthesis for P(VDF-TrFE) inks [20]. The main steps of the overall manufacturing process used by JNR to print ferroelectric sensor arrays based on P(VDF-TrFE) repeated units is illustrated in Figure 1. The fabrication of these sensing patches is done by screen-printing at a Thieme LAB 1000. A transparent and flexible (175 μm thick) DIN A4 plastic foil (Melinex^®^ ST 725 from DuPont Teijin films, USA) is used as the substrate; it ensures high flexibility and good adhesion of the functional materials applied during the screen-printing process (Figure 1a). First, the circular bottom electrodes of the P(VDF-TrFE) are screen-printed (Figure 1b). In the second step, the ferroelectric polymer P(VDF-TrFE) is screen-printed onto the bottom electrodes, followed by a short curing step at 110 °C. The curing step supports the formation of the crystalline piezo- and pyroelectric β -phase and accelerates evaporation of the solvent. Figure 1c also includes the third step of screen-printing the top electrodes. Either PEDOT: PSS or silver or carbon have been used as these top electrodes [21]: it is worth noting that the carbon layer (Figure 1d) is alternative to the usage of PEDOT:PSS or silver (Figure 1c). Conductive silver ink has been used for electrical interconnections (Figure 1e). 

A final UV-curable lacquer layer is deposited on top for overall sensor protection. The poling procedure then aligns in the thickness direction randomly oriented dipoles contained in P(VDF-TrFE) crystallites. This has been achieved by hysteresis poling of each sensor with an alternating electric field at a frequency between 2 and 10 Hz and a magnitude of 100 MV/m, corresponding to twice the coercive field strength. Final geometries of sensor array patches have been obtained through cutting the manufactured foil with a Trotec Speedy 300 laser. The full deposition process has been thoroughly presented in [8,21], to which the reader is referred for further details.

**Figure 2:**
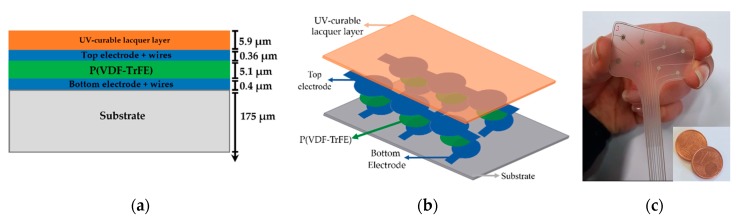
(**a**) Cross sectional view of a single sensor unit: Indicative thicknesses of the various layers have been extracted from scanning electron microscopy (SEM) pictures sent in private communications; (**b**) Sketch of the sensing patch; (**c**) Picture of a real sample.

Figure 2 depicts (a) the cross-sectional view of a single sensor unit and (b) the structure of a sensing patch built on a sensor array and (c) a photo of a sample.

#### 2.1.2. Design of the Sensing Patches

Two sets of sensing patches have been designed and manufactured. The former is intended for a textile glove with sensorized fingertips and palm, while the latter includes skin patches for the fingers and palm of the prosthetic Michelangelo Hand designed by Ottobock [17]. 

Sensor densities of the fingertips and of the palm have been oriented by psychophysical measurements of the spatial acuity of the human skin [22]. Usually to define the point-localization threshold, a stimulus is presented to the skin, followed in time by a second stimulus that may or may not be applied to the same site. Observers are required to say whether the two stimuli occur at the same or different locations. The point localization threshold is ~1–2 mm on the fingertip and around 1 cm on the palm. These values are only for reference, as the spatial acuity of the artificial skin is strongly dependent upon the thickness and on the material of the protective layer, as demonstrated in Seminara 2018 manuscript [18]. In particular, we refer to the proportionality coefficient γ plotted in [18], which gives a measure of the skin spatial acuity through the sensor receptive field, i.e., the spatial concentration of the mechanical stress information around a single sensor. The γ coefficient depends on the thickness of the elastic cover layer, and vanishes at a distance between the point force and the sensor axis, that marks the transition to the region where the force does no longer affect the given sensor. 

**Figure 3:**
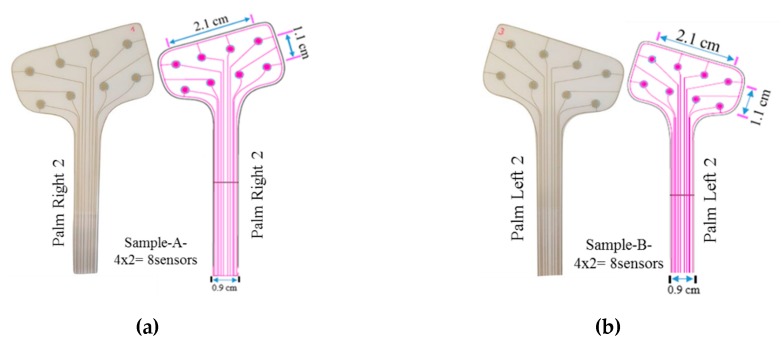

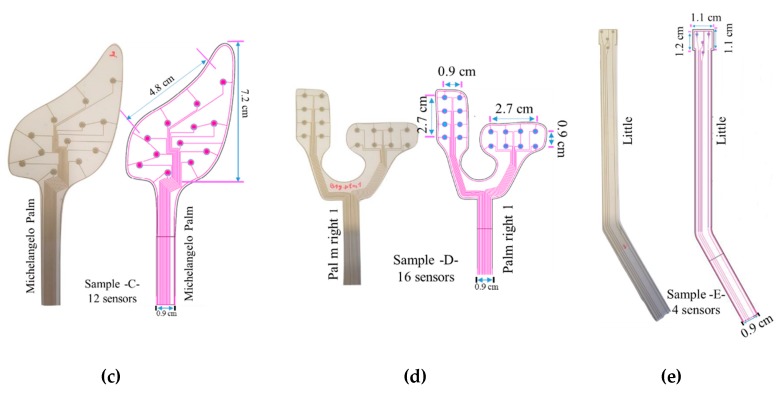
Design of different sensing patches. (**a**) Sample A: Palm right 2 - 4 × 2 array = 8 taxels, Taxel diameter = 2 mm, center-to-center pitch = 1 cm, total sensing area = 2.1 × 1.1 cm^2^. (**b**) Sample B: Palm left 2 − 4 × 2 array = 8 taxels, Taxel diameter = 2 mm, pitch = 1.1 cm, total sensing area = 2.1 × 1.1 cm^2^. (**c**) Sample C: Michelangelo palm −12 taxels, taxel diameter = 2mm. (**d**) Sample D: Palm right 1 − two 4 × 2 arrays −16 taxels, Taxel diameter = 2 mm, center-to-center pitch = 0.9 cm, total sensing area = 0.9 × 2.7 cm^2^. (**e**) Sample E: Michelangelo little - 4 taxels, taxel diameter = 1 mm, total sensing area: square side =1.1 cm.

Five different patch geometries have been experimentally characterized, and the correspondent results are presented in the current article. The patch layouts are shown in Figure 3.

### 2.2. Experimental Setup

**Figure 4:**
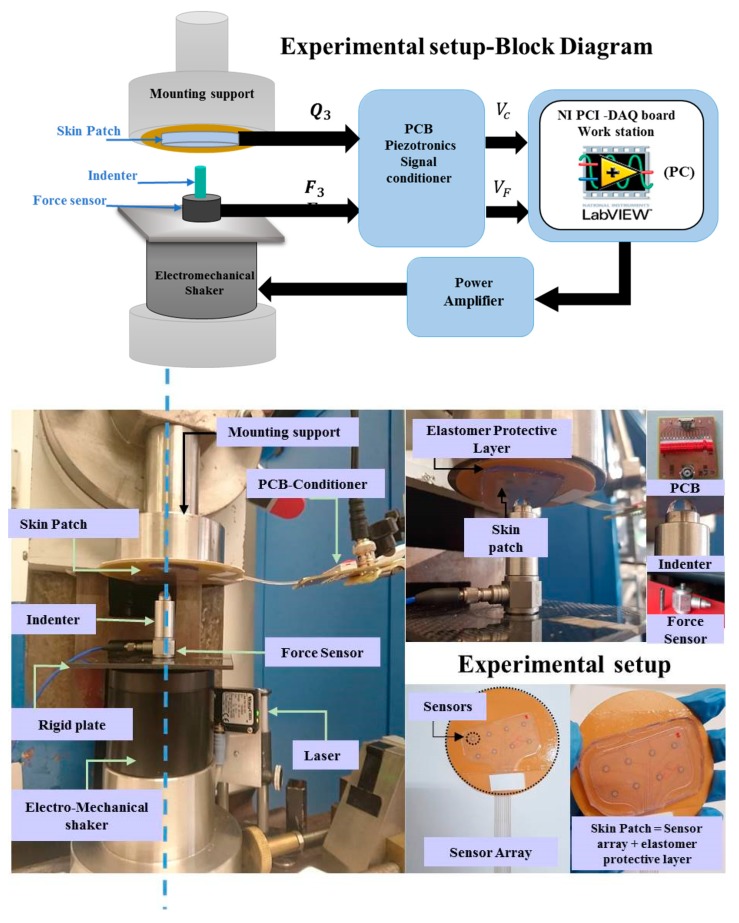
Experimental setup. Top: Block Diagram, Bottom: Pictures of the setup. The blue dotted line shows the alignment of the testing elements.

Twelve skin patches of five categories (the A, B, C, D and E samples, as shown in Figure 3) were tested using the mechanical chain shown in Figure 4 and described in Seminara’s manuscript [18]. Each sensing patch was integrated on a rigid substrate and covered by an elastic protective layer, thus building a skin patch (see the bottom part of Figure 4). In particular, the same elastomer material has been used for stress transmission as in [18].

**Figure 5:**
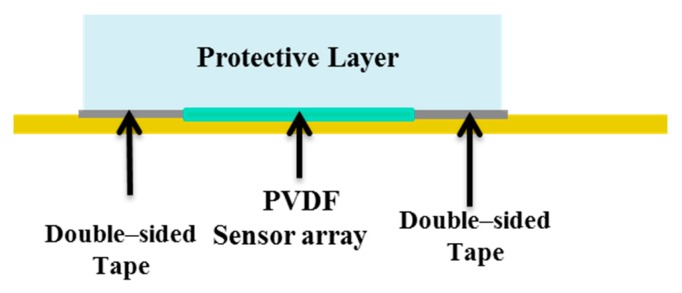
The applied coupling scenario.

Building a skin structure that mimics, as close as possible, the conditions imposed by the model presented in [18] has two implications. On the one hand, we would like to enable sensors to work in a pure compressive mode. This would require that the coupling does not lead to the development of normal stresses T_1_ and T_2_ in the sensors which are comparable to T_3_. Operationally, in order to be able to keep the sensing patch intact for use after the validation stage, we have simply laid it over a rigid substrate with no further mechanical constraints (for better clarity, see Figure 5). This implies that the boundary conditions at the contact sensing patch, the rigid substrate, would be a simple roller. On the other hand, the upper protective layer is kept in contact with the substrate, constraining the lateral boundary of its bottom surface with double-sided adhesive tape (Model 3M300L, 3M). This scheme allows one to assume a roller type boundary condition at the elastomer at the bottom with constrained boundaries. The applied coupling scenario is illustrated in Figure 5.

A rigid plate was fixed on the moving head of an electromechanical shaker (Brüel and Kjaer, Minishaker Type 4810 from HBK company, Germany). A rigid spherical indenter (R = 4 mm) and a piezoelectric force transducer (Model 208C01, PCB Piezotronics, MTS system, were coupled on the upper head of the rigid frame. The skin patch assembled on the rigid circular plate was then mounted on a fixed support and faced down side. 

During the tests, we applied a mechanical input (force) and measured the electrical output (charge). A preload was first applied to guarantee indenter-PDMS contact during the whole mechanical stimulation. The value of the preload has been controlled by a laser (Waycon LAS TM10), allowing us to fix the displacement of the rigid plate at a certain value for a certain preload, through displacement–force calibration curves. A swept sine signal was provided to an electromechanical shaker by a graphical user interface (GUI) developed with NI LabVIEW on a host PC and NI DAQ data acquisition board. The signal was amplified using a Power Amplifier (Type 2706). All of these elements have been accurately aligned before any test. Forces in the frequency range of (0.5–1 kHz) have been applied through the spherical indenter shown in Figure 4 and coupled to the electromechanical shaker. The force transducer (stimulus) and the charge developed by the sensor (response) were conditioned by PCB Sensor Signal Conditioner (482C54) and processed in frequency to give the System Response Function (FRF) at each frequency step. We recall that FRF corresponds to the ratio between the Fourier transform of the output charge and that of the input force. 

### 2.3. Reference Skin Structure and Model

**Figure 6:**
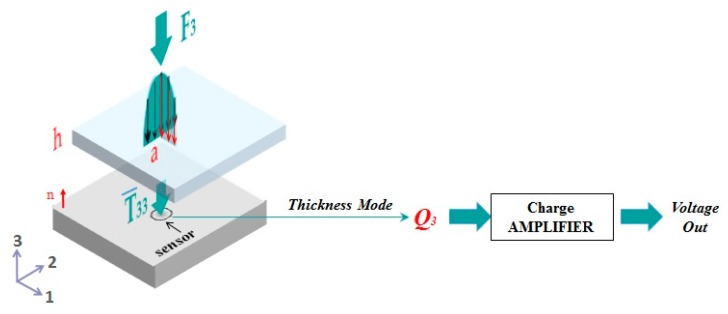
Sketch of the general working mechanism of the P(VDF-TrFE) sensor: The Hertzian input force (with contact radius *a*) is transmitted to the sensor (with radius r_T_) through the elastomer layer of thickness *h*. With the presupposition that the sensor works solely in compressive mode, it directly converts the received normal stress T_3_ into electrical displacement D_3_, through a characterizing piezoelectric coefficient, namely the d_33_ (1). A charge amplifier is used to convert the total sensor charge into voltage.

As mentioned in the introduction, in order to validate sensor behavior without damaging the sensors themselves, the sensing patches need to be integrated into a rigid substrate and covered by an elastomer. Hence, the indenter force is applied to the surface of the protective layer and transmitted to the sensors, working in thickness mode. In order to derive the stress acting on the sensor, our previous model [21] has been used, and is briefly summarized below (Figure 6). The ultimate use of the model is to estimate the electrical sensor output (i.e., charge) from a measure of a basic mechanical action at the skin surface. In other words, using the constitutive relationship of the sensors working in thickness-mode (purely compressive), one might write:

(1) $$Q_{3}=\pi r_{T}^{2}d_{33}{\bar{T}}_{3}$$

where Q_3_ is the total sensor charge measured by the charge amplifier [23], $r_{T}$ is the sensor radius, d_33_ is the P(VDF-TrFE) piezoelectric coefficient and ${\bar{T}}_{3}$ is the normal stress component T_3_ averaged over a single sensor. 

The application of the model leads to the following relationship between the charge and the applied force F_3_:

(2) $$Q_{3}=-d_{33}\frac{3}{2}{\left(\frac{r_{T}}{h}\right)}^{2}\;\sigma \;(\frac{a}{h},\frac{r_{T}}{h})\;F_{3}$$

where *h* is the elastomer thickness, *a* is the contact radius and σ is an output function of the theory, expressed as a double integral to be solved numerically, for each chosen value of $\frac{r_{T}}{h}$ and $\frac{a}{h}$.

The radius *a* of the imprint is related to the applied load F_3_ by the equation [24]:

(3) $$a^{3}=\frac{3F_{3}R}{4E}(1-\nu^{2})$$

where R is the indenter radius, and E and ν are Young’s modulus and the Poisson ratio of the elastic protective layer, respectively. Note that the given preload affects the contact radius *a* (3), while the amplitude of the dynamic swept sine force determines the PVDF charge. On the contrary, the dynamic component does not affect the computation of the contact radius, as the dynamic signal amplitude is negligible with respect to the preload. 

For a given skin geometry, associated with a specific $\frac{r_{T}}{h}\;$, (2) allows one to estimate the effective piezoelectric coefficient d_33_ of each P(VDF-TrFE) sensor, once the charge Q_3_ and the (normal) applied force F_3_ centered on that specific sensor have been measured. Comparison with the expected value of d_33_ [8,21] helps validating sensor functioning. 

The effect of the finite thickness of the elastomer layer has been expressed by the value of sigma for the given skin geometry presented in this paper and calculated numerically through FEM simulations, as discussed in Seminara’s manuscript [18]. In particular, we considered an elastic, virtually incompressible, medium (Poisson ratio sufficiently close to 0.5) consisting of a layer of finite thickness h = 2.5 mm, length l = 40 mm and width b = 20 mm. Length and width of the layer have been chosen arbitrarily, with the sole requirement of the distance between the elastomer side and the sensor center being much larger than the sensor radius, such as to justify the assumption that the lateral boundaries do not significantly affect the stress field acting on the sensor. 

The elastomer surface is presumed to be subjected to an external Hertzian pressure distribution, the contact radius *a* being dependent from R, F_3_, E and ν, as for (3). The indenter radius R is 4 mm in all the present study, the employed value for the elastomer modulus E is the result of the experimental characterization of the elastic layer reported in [18] and corresponds to 16 [MPa] (slope of the linear portion of the stress–strain curve), while ν is assumed to equal 0.5. 

As said above, the contact radius is mainly a function of the given preload, as the dynamic signal amplitude is negligible with respect to the preload itself. As discussed in the previous section, a roller type boundary condition was assumed at the lower boundary, while the perimeter is constrained.

**Figure 7:**
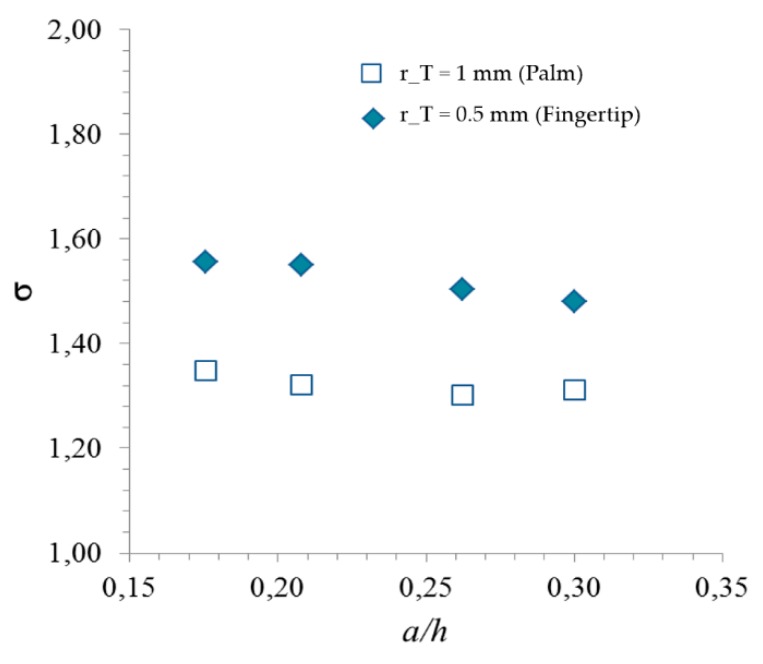
Results for the numerical COMSOL simulations for the finite case. The proportionality coefficient σ between average normal stress T_3_ on the sensor and overall (Hertzian) contact force F_3_ (2) is plotted versus the imprint radius a (contact size) scaled by the elastomer thickness h. Note that the applied force is centered on the sensor. The two curves are associated to two different sensor sizes: r_T_ = 1 mm (sensors on the palm), r_T_ = 0.5 mm (sensors on the fingertip).

The proportionality coefficient sigma, which allows to estimate the d_33_ value of each sensor (2), based on the measured ratio between Q_3_ and F_3_, is reported in Figure 7. The value of the contact radius *a* changes with the following preload values: PL = 0.6, 1, 2 and 3 N. It is worth pointing out that the present results are consistent with those found in Seminara’s work [18]. As well, note that values of σ obtained for palm sensors differ slightly from the fingertip ones, as σ depends on the ratio $\frac{r_{T}}{h}$ (recall (2)).

In addition, we have verified the consistency of the experimental setup for the sensing patch with the pure compressive mode assumption. Then, we have performed a series of simulations aiming to evaluate the stress tensor in the sensing patch as a function of the preload, subject to a roller type boundary condition at the bottom and free lateral boundaries. These simulations show that the normal stresses T_1_ and T_2_ keep at least an order of magnitude smaller than T_3_ within the sensing patch. Recalling the complete constitutive relationship [7]:

(4) $$D_{3}=d_{31}T_{1}+d_{32}T_{2}+d_{33}T_{3}$$

and noting that d_31_ and d_32_ are smaller than d_33_ [7], we end up concluding that the assumption of pure compressive mode was sufficiently adequate for the experimental setting.

## 3. Results

### 3.1. Morphology of the Sensing Patches: Issues

All sensing patches have been visually inspected using first a photo scanner (EPSON perfection V800 photo) and then an optical microscope (Nikon eclipse LV100 and Wild M32).

**Figure 8:**
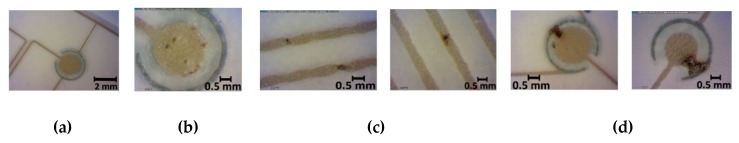
(**a**) Normal sensor, (**b**) Fault in the sensor top electrode (pole), (**c**) Cut in the sensor tracks, due to short circuits during the poling procedure, (**d**) Shortcuts between sensor tracks.

**Figure 9:**
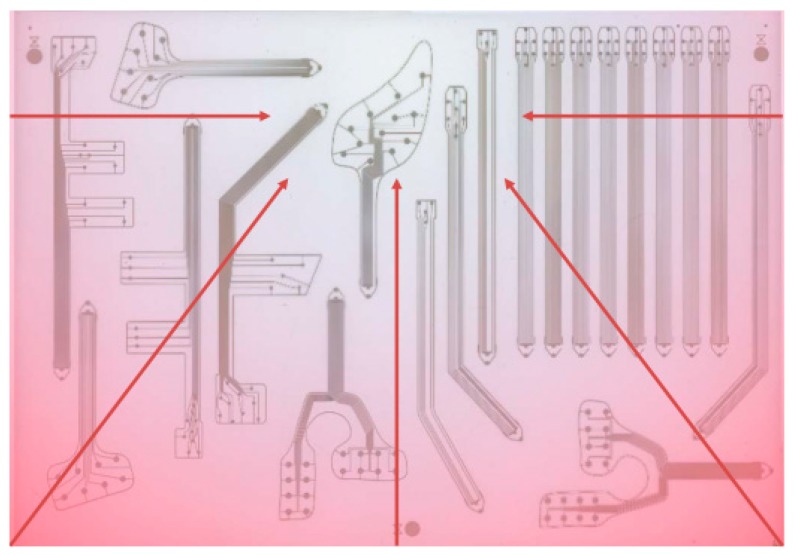
The heat map of the fabrication substrate (DIN A3) prone to shrinkage.

Some fabrication defects have been detected (see Figure 8). They are listed below:

1. *Faults in the top sensor electrode.* The choice of silver ink for the top electrode has been the result of a compromise between resolution, conductivity and top-electrode performance. Using silver, the printing resolution was very good and the conductivity was very high at the 100 °C temperature treatment. At a careful examination, small defects were detected, due to solvents in the ink (Figure 8b). However, this does not heavily compromise sensor behavior.
2. *Interrupted tracks* (Figure 8c). During high-voltage hysteresis poling, sensor lines burned for current exceeding a given threshold due to short circuits between top and bottom electrodes (caused by their too small distance).
3. *Short circuits.* They occurred between sensor lines or due to the misalignment between top and bottom electrodes (Figure 8d). The high spatial resolution led to too small distances between lines and top/bottom electrodes, causing short-circuits due to the shrinkage of the whole DIN A3 fabrication substrate during high temperature treatment. Figure 9 shows the heat map of the substrate prone to shrinkage. We observed that certain sensing patches (such as M-Palm) lie on the blue sweet spot, corresponding to less shrinkage. This guarantees a larger number of working sensors. Other samples (such as palm right 2) are on the red zones, associated with high shrinkage. This causes higher number of short circuits, which in turn leads to less working sensors than expected.

In summary, the required high resolution (i.e., small sensor size, short distance between the top and bottom electrodes, short distance between the sensor tracks) is challenging. In particular, such fine structures cannot be distributed over such a large area (DIN A3) if the substrate is not dimensionally stable during all process steps (including sensor polarization). How these fabrication defects affected sensor behavior is illustrated in Section 3.1. 

### 3.2. Experimental Tests

A series of experiments were conducted to extract the sensor behavior, i.e., ultimately their d_33_ values, from indentation tests on the skin surface, by using the model illustrated in Section 2.3. Before running each test, a preload has been applied to guarantee indenter-skin contact during the entire mechanical stimulation. As specified in Section 2.3, the preload is responsible for determining the contact radius *a* (3), as for all tests the amplitude of the dynamic oscillation is maintained low enough (F_dyn = 0.09 N) not to significantly affect the contact area. 

Different P(VDF-TrFE) sensing patches have been tested as described in Section 2.2. We applied a swept sine signal from 0.5 Hz up to 1000 Hz by the electromechanical shaker at each sensor epicenter on the e-skin outer surface, causing e-skin indentation aligned with each sensor center. We recorded the sensor frequency-response function one-shot over the whole frequency range. The numerical model described in Section 2.3 has been integrated into the LabVIEW software, directly giving the frequency behavior of the d_33_ piezoelectric modulus (both real and imaginary parts) of each solicited sensor, calculated from the sensor frequency response function as for (2). Sigma values have been extracted from Figure 7, each time in accordance with the specific preload and sensor radius. 

#### 3.2.1. Frequency Range Selection

In a preliminary stage, we investigated the minimal value of the applied preload that ensured a stable behavior of d_33_. Multiple tests at preloads less than 1 N have been run over the whole frequency range (0.5 Hz–1000 Hz), especially at preload 0.6 N. Main observation is that this low value for the preload does not ensure a stable contact during oscillations of the indenter over the skin patch, due to the dynamic amplitude of the indenter oscillation being not enough smaller than the preload itself. This causes noisy behavior for the d_33_. For that reason, in the rest of the study, the results at this preload are not reported. 

**Figure 10:**
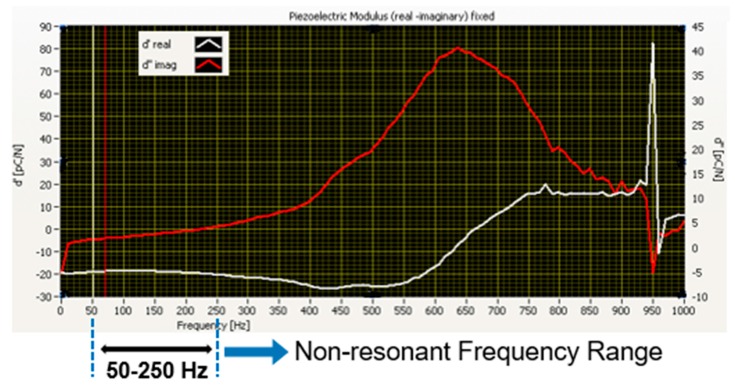
Example of the frequency behavior of both the real (white curve) and imaginary (red curve) part of the d_33_ piezoelectric coefficient. The scale for the Im (d_33_) (= d’’) is on the right *y*-axis.

Then, tests have been done at preloads 1, 2 and 3 N. It turned out that resonances do exist, and their characteristic frequencies depend upon the preload. In the 300–750 Hz range, a systematic preload-dependent resonance peak is responsible for sign flipping of the real part of the d_33_ coefficient. At low preloads (i.e., PL = 1 N) the resonance falls in the 300–500 Hz range, while at higher preloads (i.e., PL = 2, 3 N) the resonance shifts to the 500–750 Hz frequency range. Around 950 Hz, a mechanical resonance appears due to high vibrations from the shaker system while stopping. As reported in Seminara’s work [18], resonances may derive from a variety of causes (e.g., movable contacts, contact surface asperities, motor-induced vibrations), which cannot be reliably controlled. The model can only be applied with dynamic contacts with forcing frequencies that fall outside the range of any notable resonance [18]. Therefore, a non-resonant 50–250 Hz frequency range has been identified, where the frequency response function is systematically quite flat. In particular, the imaginary part of the d_33_ piezoelectric coefficient, which accounts for any viscoelastic component of the response, is systematically roughly an order of magnitude smaller than the real (elastic) part. The aforementioned statements are clarified in the representative example reported in Figure 10, where both the real and imaginary parts of d_33_ are expressed as a function of frequency, and the non-resonant range is highlighted.

Based on these results, hereafter the imaginary part of the d_33_ coefficient will be ignored and “Re” will be removed from the notation. In other words, the system is treated as purely elastic. Moreover, each run has been performed, stimulating the skin over the whole frequency range, yet the corresponding d_33_ response is averaged over the non-resonant range only.

#### 3.2.2. Systematic Sensor Validation

Each sensing patch has been tested by stimulating the e-skin surface with the same indenter (R = 4 mm) aligned with the epicenter of each selected sensor. As mentioned in Section 3.2.1, each run has been performed at small force amplitude (F_dyn = 0.09 N), and the corresponding d_33_ response has been averaged over the non-resonant range to get a single value of that coefficient for each sensor. 

**Figure 11:**
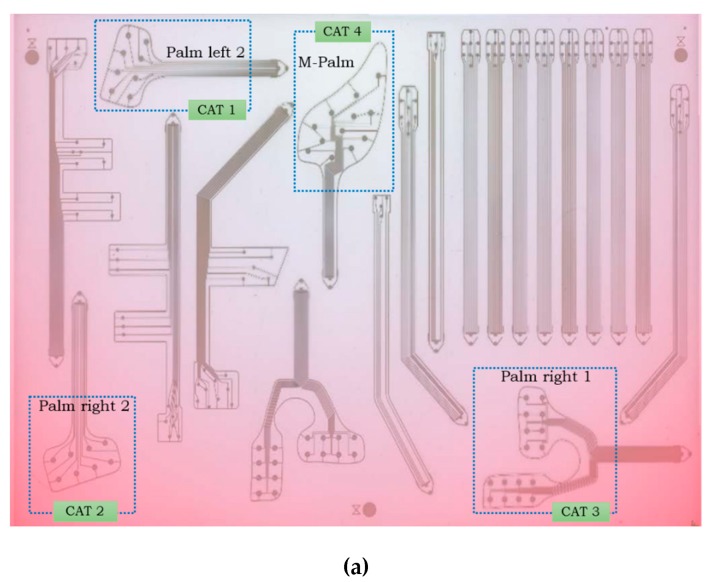

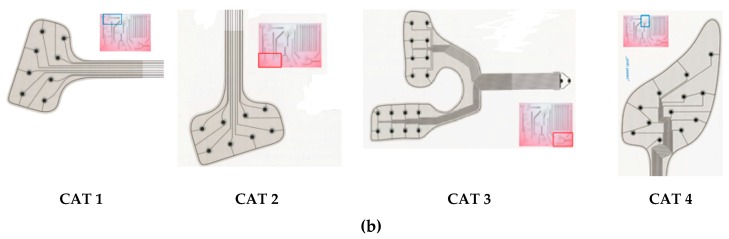
Compared categories (CAT1, CAT2, CAT3 and CAT 4) and heat map on the A3 fabrication substrate.(**a**) Heat map of the fabrication and the selected categories for testing, (**b**) Zoom in view for each category’s location.

Two sets of data have been obtained. The former data set (96 sensors in total, 10 different samples, four categories of patches) focuses on Palm sensors (i.e., sensors with diameter = 2 mm, belonging to arrays designed to cover the palm), all tested at different preloads (Figure 11). 

On the other hand, the second data set (eight sensors, two samples, Michelangelo little) focuses on finger sensors (i.e., sensors with diameter = 1 mm, belonging to arrays designed to cover the fingertips). One-way analysis of variance (ANOVA) and Tukey–Kramer’s honestly significant difference (HSD) test, for the post-hoc pairwise comparison, were used to test the statistically significant difference in the mean performance among the tested conditions. 

##### First Analysis: Palm Sensors

We selected four palm patch designs that vary in their positions and sensor number. These designs have been classified into four categories as reported in the Table 1 below.

Figure 11 illustrates how these categories are distributed over the A3 substrate used for patch fabrication. A comparative study has been performed to examine whether the shape and position over the A3 fabrication substrate affected the sensor behavior at different preloads. 

**Figure 12:**
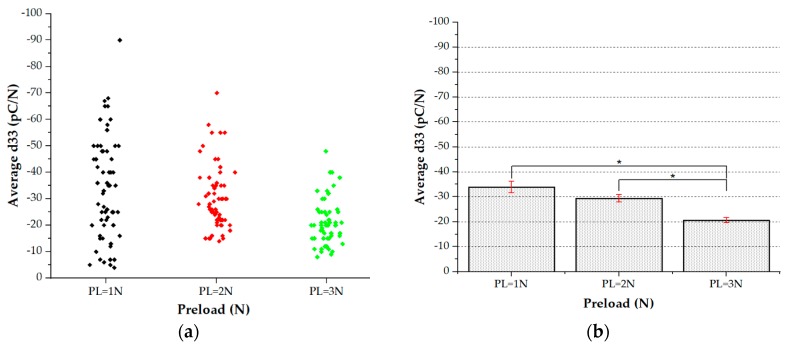
(**a**) Cloud distribution of working palm sensors. (**b**) Statistical study: One-way analysis of variance (ANOVA) (*p* < 0.05), average d_33_ vs. preload.

67 sensors out of the whole set (96 sensors) have been selected, eliminating sensors that did not work due to fabrication failures (see Section 3.1) and few sensors that gave physically unacceptable values for d_33_. Note that the number of malfunctioning sensors was quite high for this first fabrication batch, due to those issues discussed in Section 3.1. Figure 12 shows the cloud distribution of the averaged d_33_ values for the palm sensors.

All categories have been analyzed in order to check whether any dependence of the patch behavior on the specific category existed. This was needed to understand if a specific patch position affected sensor behavior, e.g., due to not uniform polarization or other unwanted effects related to the shrinkage of the substrate during the fabrication process.

**Figure 13:**
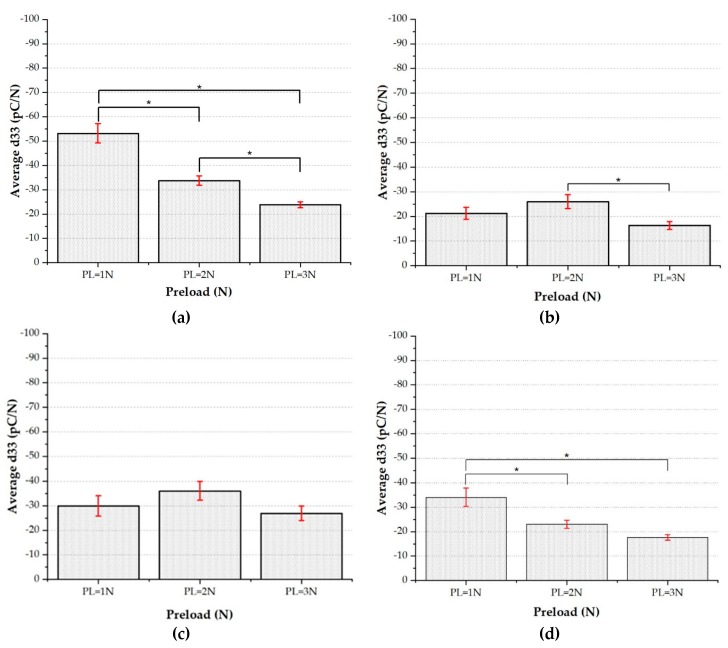
Average d_33_ vs. Preload for four different categories: (**a**) Category 1 (palm left 2). (**b**) Category 2 (palm right 2). (**c**) Category 3 (palm right 1). (**d**) Category 4 (Michelangelo palm). One-way ANOVA (*p* < 0.05) and Tukey–Kramer’s HSD test have been applied for statistical analysis.

The results presented in Figure 13 show that indeed sensor response to preload does significantly depend on the category, which is associated to a specific position on the substrate. 

In particular, note that results for categories 2 (Figure 13b) and 3 (Figure 13c) show a dependence of d_33_ on the preload, which turns out not to be statistically significant. It is worth pointing out that categories 2 and 3 are those located in the red zone of the heat map, where strong substrate shrinkage occurred. In order to check the effectiveness of the sensor fabrication technology, we have then decided to discard results referring to categories 2 and 3. 

**Figure 14:**
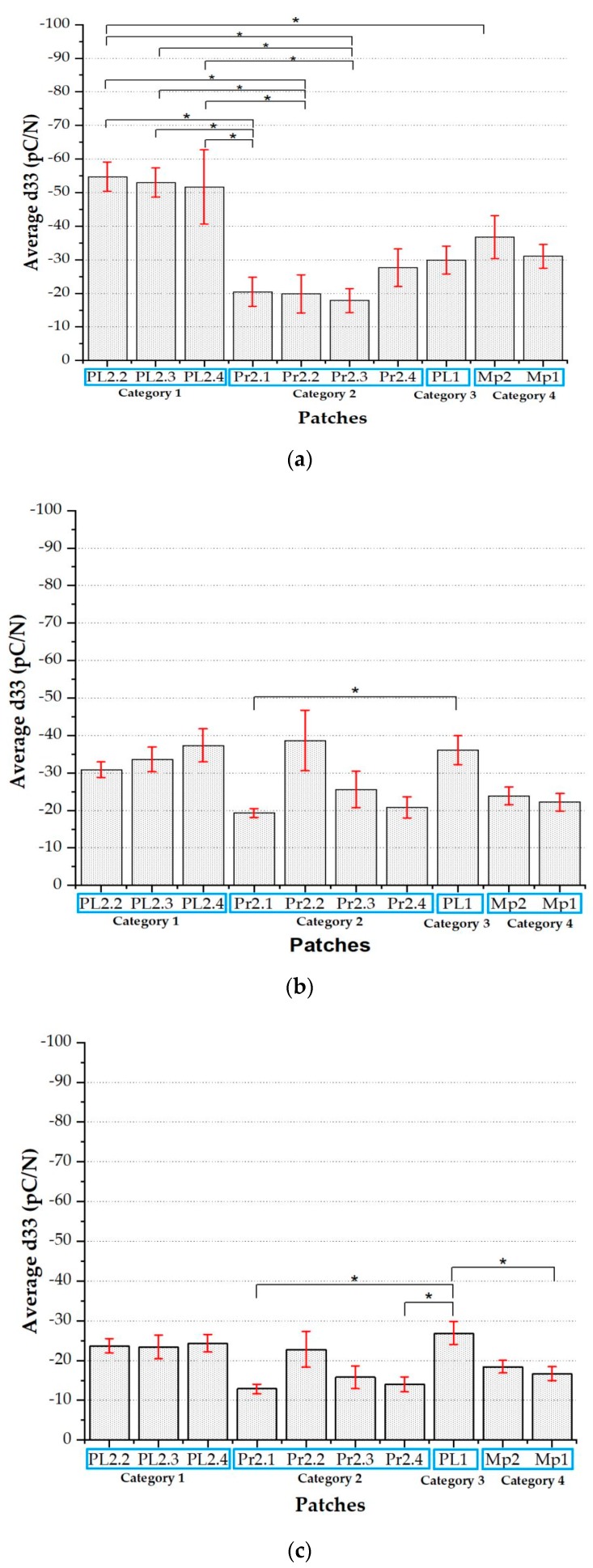
Average d_33_ vs. patches at PL = 1 N, 2 N, and 3 N, arranged respectively as (**a**), (**b**) and (**c**). The four categories and all corresponding patches can be distinguished on the *x*-axis.

On the other hand, it is reassuring to note that, as shown in Figure 14, patches belonging to the same category (including those in the red zone) are statistically equivalent among themselves, a result which does suggest the reproducibility of the fabrication process for each patch. 

**Figure 15:**
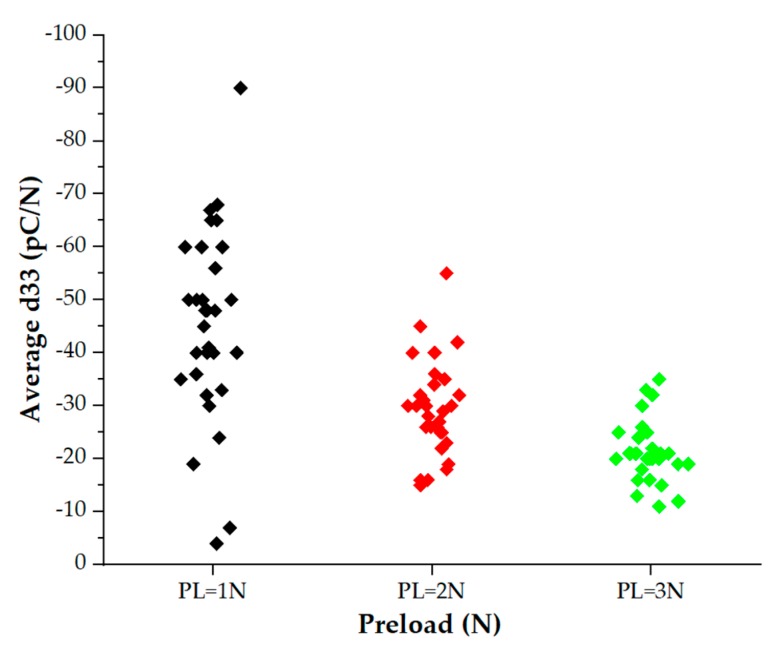
Average d_33_ vs. preload. Sensors belonging to categories 1 and 4, only. To avoid dot superposition, values associated with the same preload are plotted such that dots do not lie on the same vertical line.

**Figure 16:**
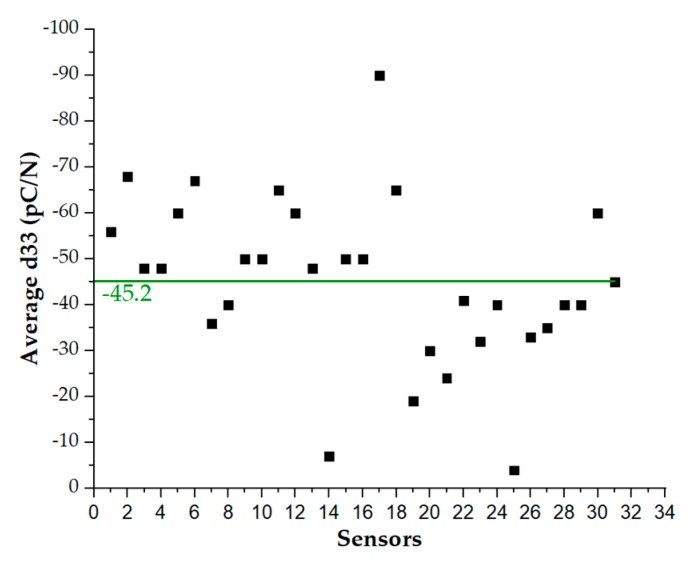

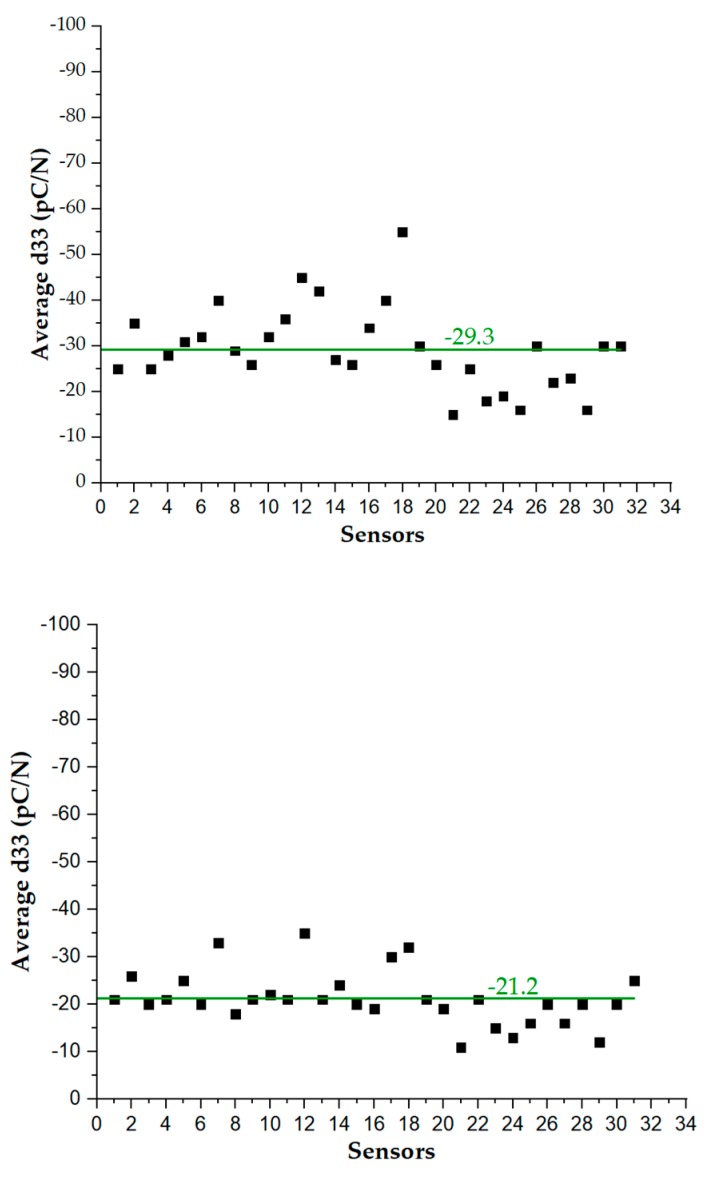
Average d_33_ for each sensor at PL = 1 N (**top**), 2 N (**middle**) and 3 N (**bottom**).

Results for all sensors belonging to the two categories located in the sweet spot associated with low shrinkage (i.e., categories 1 and 4) are plotted in the Figure 15 and Figure 16. They show d_33_ values mostly compatible with the state of the art [8].

It turns out that as the preload increases, the average d_33_ decreases, and values for different sensors exhibit a lower dispersion. In Figure 16, a best-fit line is used to compute the average of the d_33_ values associated with all sensors. Data related to the highest preload (=3 N) are well fitted using a d_33_ value equal to approximately −22 pC/N, while data corresponding to the lower preload (=1 N) yield a d_33_ value of approximately –46 pC/N. 

**Figure 17:**
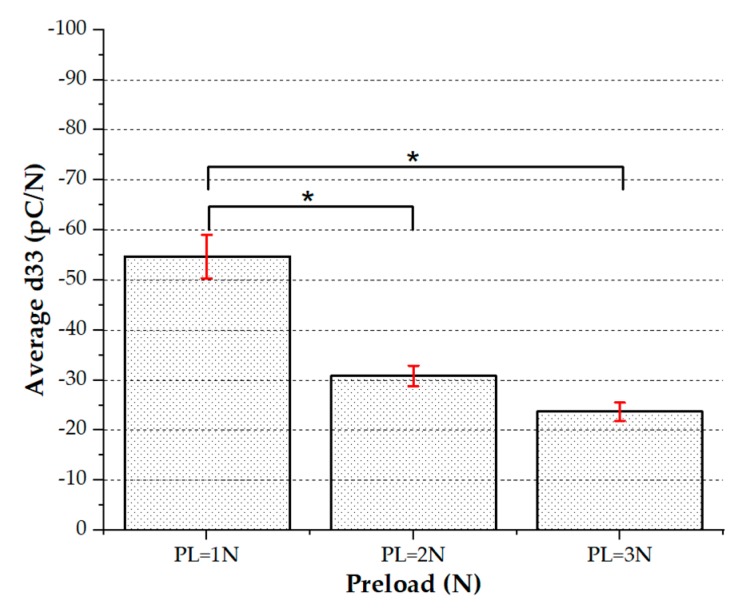

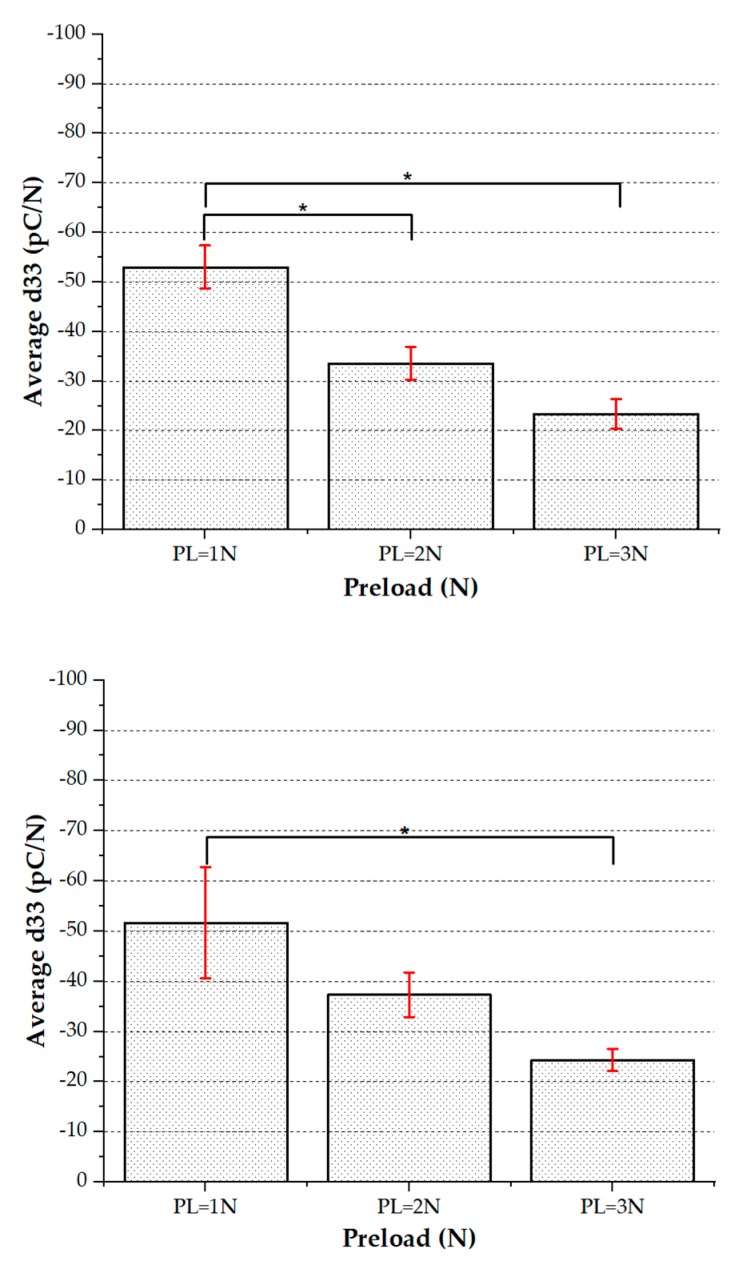
Average d_33_ vs. preload for the three analyzed patches belonging to category 1: Palm left 2.2. (**top**), Palm left 2.3 (**middle**) and Palm left 2.4 (**bottom**).

To conclude results on the first batch, we have performed a more detailed analysis of results obtained for category 1, analyzing the behavior of each patch belonging to that category. Results are plotted in Figure 17, which shows a statistically significant systematic decrease of the d_33_ coefficient with a preload for all three patches. 

##### Second Analysis (Preliminary): Finger Sensors

**Figure 18:**
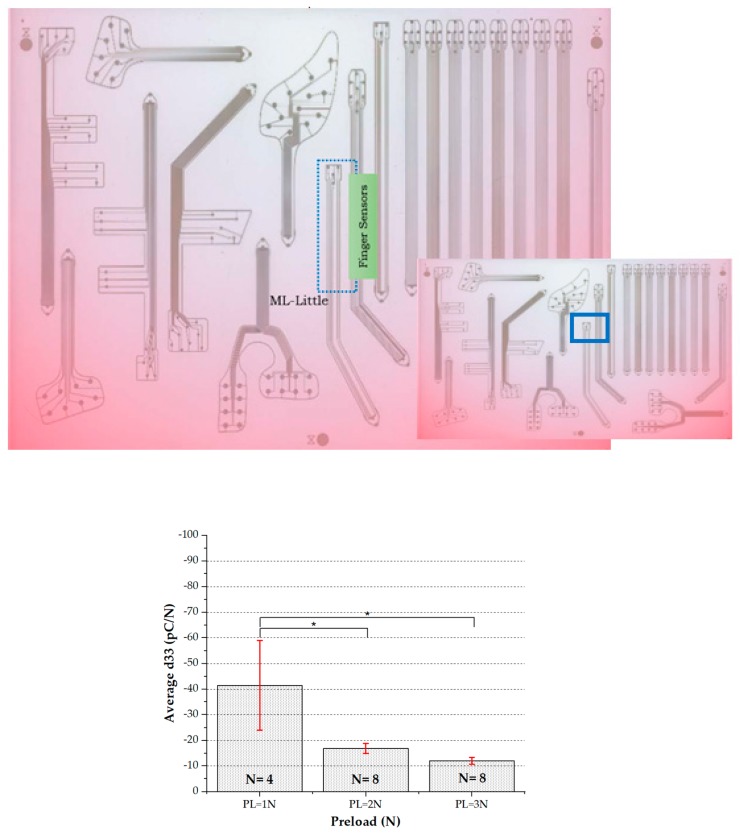
**Top**: Finger sensors (Michelangelo Little, ML) located in the sweet spot on the heat map of the A3 fabrication substrate. **Bottom**: Average d_33_ vs. preload for the two samples of Michelangelo little: ML.1 and ML.2. Statistical study: one-Way ANOVA (*p* < 0.05).

A complementary case study has been performed in order to check whether the proposed method could be extended to sensors with lower diameter (i.e., finger sensors) or not. To this aim, a second data set only including finger sensors was analyzed. It is worth remarking that the alignment procedure was particularly critical in this case: the small sensor size would require an alignment system to more precisely align the indenter with the sensor center for reliable sensor characterization using the current model. This is the reason why this analysis has been only performed on a low number of sensing patches. Two samples of Michelangelo little finger located on the sweet spot (see Figure 18-Top) were tested using the experimental setup and method reported above. Each sample has four taxels with 1 mm diameter each. Figure 18 shows the analyzed results after applying one-Way ANOVA using Tukey–Kramer’s HSD test. As for the palm, the results indicate a significant statistical difference of d_33_ at different applied preloads and a systematic decrease of d_33_ at increasing preload. 

## 4. Discussion

Table 2 shows a conclusive summary of the findings which emerged from the different experimental studies performed. A broad outcome of the performed tests is that both palm and finger sensors share the same statistically significant systematic decrease of d_33_ with preload. In the first analysis, for all categories, the patches are statistically equivalent among themselves when belonging to the same category, which proves the reproducibility of the whole deposition process. Excluding categories located in the red zone (i.e., CAT 2 and CAT 3) of the heat map, which is associated with high shrinkage, the single sensors belonging to the other two categories (CAT 1 and CAT 4) show a piezoelectric behavior (i.e., d_33_ values), which is quite compatible with the current state of the art [11]. On the other hand, the behavior of the d_33_ versus preload for CAT 2 and CAT 3 in the red zone shows no alignment with the decreasing behavior observed for patches located in the sweet spot. This result is a hint at the need of employing smaller fabrication substrates in future e-skin manufacturing, in order to considerably reduce red zones, which are not compliant to the expected sensing behavior.

Focusing on categories located in the sweet spot, all analyzed patches belonging to categories 1 and 4 have quite systematic decreasing behavior for d_33_ vs. PL. This has been checked using one-way ANOVA for statistical analysis and Tukey–Kramer’s HSD test for the post-hoc pairwise comparison. Systematically, average d_33_ behavior at PL = 1 N is statistically different from that at PL = 3 N, both for the two categories (Figure 13a,d) and for single patches from category 1 (Figure 16). This would be compatible with a non-linearity of d_33_ with respect to the preload, and with some nonlinearity in the stress–strain curve observed for this elastomer layer around 2 MPa [21]. In the second analysis, similar results were obtained for the finger sensors, despite the high distribution error, which emerges from the low number of sensors tested. 

The dispersed behavior of d_33_ (i.e., sensor response) does depend on both the fabrication process (including deposition and assembly) and on the alignment of the indenter with the sensor center. A laser-like positioning system could be used in the future to align the indenter precisely, thus avoiding errors due to wrong positioning. As for the fabrication process, these errors are the results of different factors including different point-to-point values for the sensor radius and/or for the local layer thickness and inhomogeneity in PVDF film polarization. These combined factors are considered intrinsic in the whole fabrication process, and could not be decoupled in the proposed tests.

**Figure 19:**
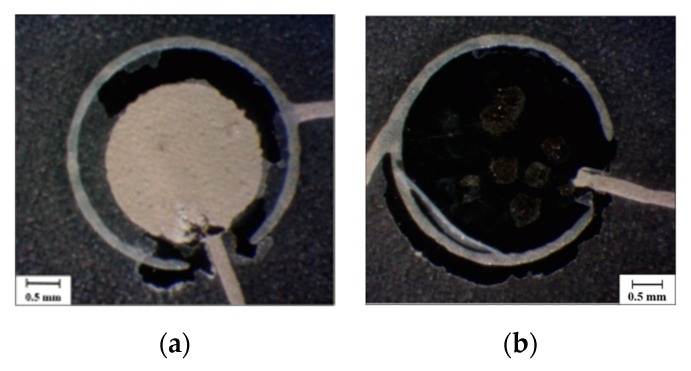
Sensor electrodes have been severely damaged after coupling with adhesive tape all over the skin patch. (**a**) An overall adhesive tape coupled sensor, (**b**) Impaired top electrode of sensor after removing the adhesive tape.

In Section 2.2, we described how we coupled the sensing patch to the substrate and to the protective layer, to be able to test sensor behavior without damaging the sensors themselves. Applying double-sided adhesive tape all over the sensors in the validation stage is not feasible unless the cover layer is the final layer, because sensors would be damaged during tape removal (Figure 19). 

It would be also better to avoid the adhesive tape between the substrate and the sensors themselves, as damages may occur during tape removal. 

Therefore, the choice of the coupling procedure is somehow obliged in the validation stage. Operationally, as described in Section 2.2, we placed double-sided adhesive tape around the sensing patch (Table 3 solution1), to rigidly couple to the substrate the protective layer on its boundaries, thus keeping in place the sensing patch itself. We also proved through simulations that this configuration leads to negligible normal stresses other than T_33_, thus confirming that sensors work in thickness mode, as required by the model.

However, this coupling procedure can only be used in the validation stage, as discussed in the following. In real applications shear contact forces on the skin surface will be possible, which requires using a real rigid coupling between the sensing patch and both the cover layer and the substrate (Table 3 solution 2), to avoid any sliding due to shear forces. This is achieved in practice by using an adhesive layer below and all-over the sensing patch itself. Care would only be needed during tape integration as non-uniform stress transmission and sensor bending can be naturally induced by the inclusion of air bubbles into the coupling adhesive layer. An underestimation of the d_33_ value is expected due to the addition of deformable adhesive layers between the sensor and both the substrate and the cover, which are not accounted for in the model. This leads not to be perfectly compliant with the model, as normal stresses other than T_3_ may contribute to the measured charge: preliminary simulations confirmed this prediction and hint at a contribution of normal T_1_ and T_2_ stresses, which is not negligible with respect to the normal T_3_ component. New models and more extensive simulations will be thus needed to describe the real application system. 

A time-saving protocol for future sensing patch validation can be extracted as an outcome of the analysis presented in this manuscript. It could be summarized as follows. As a first step, the sensing patch is to be coupled to the rigid substrate by only applying double-sided adhesive tape around the patch perimeter (Table 3 solution 1). As shown in Table 3 solution 1, the protective layer can then be applied on top of the sensing patch, being rigidly coupled to the substrate through the double-sided adhesive layer. After mounting the skin patch built as such along the mechanical chain illustrated in Figure 4, the indenter is to be aligned with a reference sensor. A laser positioning system would facilitate such a procedure, thus reducing the dispersion of sensor behavior. Avoiding complete systematic measures at different preloads, which are not needed if the scope is a check of the sensor manufacturing process, an indentation test over the non-resonant frequency range (50–250 Hz) can be quickly run at an average preload (i.e., =2 N). This procedure lasts no more than a few seconds. The indenter is then released and moved over a distant sensor, to avoid artifacts due to the relaxation of the protective layer after indenter release. The same procedure as before is performed, consisting of applying the given preload, running the indentation test, releasing the indenter and moving the indenter over a distant sensor. The same scheme is applied on all sensors belonging to the sensing patch. The result of the whole procedure is a single value of the d_33_ piezoelectric coefficient for each sensor, averaged over the non-resonant frequency range. An error signal can be set up to notify if any of the sensors has a value of d_33_ which differs from the expected value by more than a previously defined tolerance. It is important to note that, except for the initial coupling procedure and first indenter centering, the rest of the procedure can be automatized, reducing to a few minutes the validation of a sensing patch built of 15–20 sensor units. 

## 5. Conclusions and Future Work

This article tackled some of the challenges related to employing electronic skin systems in real applications. In particular, this mainly requires validating the building blocks of the e-skin system, i.e., the sensing patches, and finding adequate ways to integrate these sensing patches into an electronic skin structure which also includes structural elements. Both these steps are preliminary to include the e-skin system into the target system, e.g., a glove or a prosthetic hand.

First, a set of tools is thus needed for the validation of the fabrication technology of the sensing patches. Throughout this study, a non-invasive method to validate the deposition technique of piezoelectric polymer sensors working in thickness mode has been defined and demonstrated. In particular, this paper reports the validation of the fabrication technology of flexible screen-printed sensing patches based on P(VDF-TrFE) piezoelectric polymers. This method is independent of the specific deposition technique and can cover a large number of applications requiring the employment of artificial tactile sensing through e-skin based on piezoelectric polymer sensor such as P(VDF-TrFE). 

Extensive preliminary tests with an electromechanical setup have been performed on four different patch geometries/categories for the palm and one patch geometry for the fingertips. In particular, twelve sensing patches have been characterized (10 palm patches and two fingertip patches), 104 sensors in total (96 palm sensors and 8 fingertip sensors). P(VDF-TrFE) sensors worked in thickness-mode and a protective layer has been integrated on top of the sensing patch for stress transmission and sensor protection. Dynamic skin indentation with normal force centered on each sensor has been performed, with three different preloads (1, 2 and 3 N). An average value of the d_33_ coefficient over a non-resonant frequency range has been extracted for each sensor, without damaging the sensor itself. Obtaining expected (modeled) behavior of the electrical response of each sensor to measured mechanical (normal) force at the skin surface proves that the combination of both fabrication and assembly processes was successful.

Throughout the study course, several issues were observed such as substrate shrinkage that occurred during the fabrication process, leading to shortcuts. The proposed validation and characterization provided us with cues to optimize the fabrication of the next-line batches such as choosing smaller fabrication substrates (and smaller masks, accordingly). 

The study demonstrated that for every sensing category (i.e., CAT1, CAT 2, CAT 3 and CAT 4), the sensing patches are statistically equivalent among themselves, which proves fabrication reproducibility, one of the main requirements when fabricating large volumes. 

More specifically, after excluding the sensing categories that fall in the red zone of the heat map, i.e., that have been prone to high substrate shrinkage, the remnant sensors show d_33_ values which are quite compatible with the state of art. All the sensing patches that lie in categories 1 and 4 have a systematic declining behavior for d_33_ versus preload. This in turn is compatible with the nonlinearity of d_33_ with respect to the preload and with the few nonlinearities in the stress–strain curve observed for the PDMS protective layer [18]. The same behavior was observed from tested fingertips sensors, belonging to patches that were specifically chosen as lying in the sweet spot on the heat map. 

The current paper presents an effective, repeatable and simple characterization protocol to validate the skin patches. A laser positioning system would be useful to align the indenter with the sensor center, therefore reducing errors arising from indenter misalignment, especially when testing fingertip sensors characterized by small radius. Future studies should take this into account. A critical limitation of the developed model is the inability to predict the behavior of artificial sensors in real applications, since this would require another sensor integration procedure, including double-sided adhesive layers on both sensing patch surfaces to avoid sliding. This could be done in a future work.

The usage of e-skin patches in real scenarios (e.g., biomedical applications requiring sensorized gloves or prostheses) would likely lead to film degradation and consequent P(VDF-TrFE) aging and fatigue. Estimating the piezoelectric d_33_ coefficient from the overall system response function is a practical tool to measure the reliability of e-skin degradation, whenever embedded sensors are not accessible anymore for a direct characterization. The model presented in this paper could be adapted to take into account the coupling procedure required to avoid sliding, including the deformable adhesive layers. However, measuring how the film degrades over time implies differentially comparing the current value of d_33_ to an initial value, with no influence of the wrong estimation of that absolute initial value. 

## Figures and Tables

**Table 1:** Palm patches categories (Data set 1, palm sensors).

Category	Category Name	Number of Tested Patches	Number of Sensors/Patch
Category 1	Palm left 2	3	8
Category 2	Palm right 2	4	8
Category 3	Palm right 1	1	16
Category 4	Michelangelo palm	2	12

**Table 2:** Summary of results.

Sensor Location	Study		Number of Patches	Preload	Number of Working Sensors (used for statistics)	Average *d*_33_	Standard Error (SE)	Main Result
**Palm**	**All sensors at different preloads**		10	PL = 1 N	67	–34.014	2.29304	Statistically significant systematic decrease of d_33_ with preload
				PL = 2 N		–29.492	1.47302	
				PL = 3 N		–20.656	1.00846	
	**Categories**	*Category 1*	3	PL = 1 N	18	–53.222	3.989	At all preloads, these patches are statistically equivalent
				PL = 2 N		–33.777	1.91239	
				PL = 3 N		–23.833	1.23206	
		*Category 2* *(red zone)*	4	PL = 1 N	23	–21.260	2.3983	At all preloads, these patches are statistically equivalent
				PL = 2 N		–26.043	2.83155	
				PL = 3 N		–16.347	1.58876	
		*Category 3* *(red zone)*	1	PL = 1 N	23	–29.923	4.13779	At all preloads, these patches are statistically equivalent
				PL = 2 N		–36.076	3.84218	
				PL = 3 N		–26.923	2.89885	
		*Category 4*	2	PL = 1 N	13	–34.076	3.75613	At all preloads, these patches are statistically equivalent
				PL = 2 N		–23.076	1.62694	
				PL = 3 N		–17.615	1.14656	
**Finger**	**All sensors at different preloads**		2	PL = 1 N	4	–41.5	17.46663	Statistically significant systematic decrease of d_33_ with preload
				PL = 2 N	8	–16.875	1.95884	
				PL = 3 N	8	–12.0625	1.35105	

**Table 3:** Illustration of different coupling methods.

**Solution 1: Validation stage** **Skin patch built applying double-sided adhesive tape around the sensing patch.**	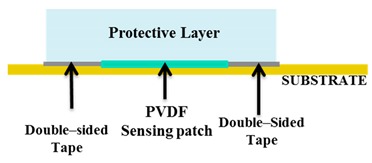
**Solution 2: Real applications** Skin patch built applying double-sided adhesive tape all over below and above the sensing patch.